# Height of Medial Longitudinal Arch in Healthy Adults Within Different Categories of BMI

**DOI:** 10.7759/cureus.93526

**Published:** 2025-09-29

**Authors:** Jaykumar D Soni, Dhriti Shah

**Affiliations:** 1 Musculoskeletal and Sports, College of Physiotherapy, Sumandeep Vidyapeeth Deemed to Be University, Vadodara, IND

**Keywords:** bmi, foot arch, height of arch, medial longitudinal arch, obese

## Abstract

Background: Alteration in the height of this medial arch increases the propensity for lower limb injuries. The aim of the current study was to identify variations in the height of the medial arch across BMI categories. Another objective of this study was to assess the variation in medial longitudinal arch (MLA) height across BMI categories using the normalized truncated navicular height (NTNH) method in a healthy adult population.

Method: We screened 360 healthy adults aged 18-45 years. We used the NTNH method to measure arch height, an index card to measure navicular height, and the footprints of the subjects to measure truncated foot length. We divided the navicular height by the truncated foot length to determine the NTNH and used the values to classify the foot arch as either low, normal, or high.

Results: Pearson’s chi-square test revealed no statistically significant correlation between BMI and the arch type of the left and right foot (p=0.097, p=0.136) or BMI and maintaining a prolonged position (p=0.212, p=0.399). However, there was a significant correlation of the left and right arch type with gender (p<0.001, p<0.001) and the type of footwear worn (p=0.008, p=0.047). The odds ratio revealed that high arches were more prevalent in the underweight group, and low arches were more prevalent in the overweight group.

Conclusion: No statistically significant correlations were found between BMI and medial arch height. However, the foot arch type correlated significantly with gender and the type of footwear worn by the subjects, though not with remaining in one position for a prolonged period. High arches were more prevalent in the underweight group, and low arches were more prevalent in the overweight group and the obese group.

## Introduction

The human foot is an ergonomically efficacious body structure. It withstands the immense pressures created by various postures and dynamic activities, provides proprioception during interaction with a supporting surface, facilitates a bipedal stance, enables orthograde locomotion, and stabilizes the body in various challenging environmental conditions [1,2]. The presence of arches in the foot is among the most remarkable morphological and functional changes in human evolution [3]. These arches, formed through the exceptional alignment of bones and soft tissue structures, permit the foot to function as a stabilizer as well as a shock attenuator and, thus, play an indispensable role in maintaining stability, sustaining the weight of the body, and dispersing the ground reaction forces produced during various activities [4,5]. Each foot has three arches: a medial longitudinal arch (MLA), a lateral longitudinal arch, and a transverse arch [6]. Of these three arches, the MLA is the highest and plays the largest role in absorbing ground reaction forces [7]. Its framework is established by the calcaneus, the talus, the navicular, the three cuneiforms, and the first three metatarsals, with the navicular bone functioning as the keystone [8].

As defined by the World Health Organization (WHO), an individual’s BMI is the weight divided by the square of height (kg/m^2^). The WHO standards categorize the BMI as follows: underweight < 18.5 kg/m^2^; normal 18.50-24.99 kg/m^2^; overweight ≥ 25 kg/m^2^; and obese ≥ 30 kg/m^2^ [9]. Obesity has been increasing worldwide because of affluent and sedentary lifestyles, decreased physical activity, and poor eating habits.

The MLA bears three times as much weight in obese people as in those with a normal BMI, resulting in mechanical and functional alterations in the foot. These changes may result in unfavorable biodynamic changes, alter the distribution of pressure, reduce stability, further limit physical activity, and lower the quality of life [10-13]. By measuring the height of the MLA in adults in the various categories of BMI and comparing this height in obese individuals with that in normal-weight individuals, researchers have found a correlation between BMI and foot arch type, though other studies have found no significant association [14-18]. The current study was conducted to help resolve this inconsistency.

This article was previously presented as a meeting abstract at the International Conference on Physical Therapy and Sports Medicine on September 21-22, 2023.

## Materials and methods

We conducted an observational cross-sectional study, collecting the data at Sumandeep University and Dhiraj Hospital in Vadodara, India. The following materials were used: blank index card, marker, ink pad, graph paper, scale, pencil, step stool, tissue paper, and isopropyl alcohol (for wiping the ink) The calculated sample size was 323 subjects, and we collected data from 360 individuals over one year. Those included in the study were healthy, willing to participate, and between 18 and 45 years of age. Both men and women were included. Patients with a history of lower limb fractures or surgeries or soft tissue injuries to the ankle or foot in the previous six months, congenital or acquired deformities of the lower limbs, neurological disorders, or any foot infection were excluded from the study. The outcome measures of the study were BMI and normalized truncated navicular height (NTNH). The intra-rater readability of NTNH was 0.98 [7].

Procedure

The study was approved by the Sumandeep Vidyapeeth Institutional Ethics Committee (SVIEC no. SVIEC/ON/PHYS/ BNPG20/D21005) and was registered with Clinical Trials Registry-India (no. CTRI/2021/05/043894).

The convenience sampling method was used. During recruitment, the study procedure was explained in detail to the participants who fulfilled the inclusion criteria in their languages.

We calculated the BMI by measuring the height and weight of the subjects. We calculated the NTNH by palpating and marking the navicular tuberosity with a blank index card aligned to the medial aspect of the foot while it was kept perpendicular to the floor. A mark was drawn on the index card at the level of the dot to indicate the navicular tuberosity. Later, the distance from the base of the index card to the level of the dot was measured in millimeters using a Vernier caliper to calculate the navicular height. Similarly, the navicular tuberosity was marked on the opposite lower limb, and its height was measured.

To obtain the participants’ footprints, we asked them to stand and press their feet on an ink pad and then place them on a piece of graph paper (mm graph: 10 squares/cm) marked with their name, age, and sex. The graph papers were then left to air dry, and the participants were asked to place their feet on a microfiber sheet to absorb the excess ink and were seated comfortably and provided with tissue paper and isopropyl alcohol to clean their soles. After the footprints on the graph paper were dry, two parallel horizontal lines were drawn on the paper with a pencil. The first line was drawn at the level of the prominent aspect of the first metatarsophalangeal joint, and the second line was drawn at the most posterior part of the heel. To calculate the truncated foot length, the distance between these two parallel lines was measured in centimeters. The navicular height was converted to centimeters and divided by the truncated foot length with the help of a calculator to derive the NTNH. The arches of the patients’ feet were then classified as follows: ≤ 0.21 low; ≥ 0.22 normal; and ≥ 0.32 high.

## Results

We analyzed the data that we collected using SPSS software version 21.0, expressing them as mean ± SD for the NTNH in the categories of BMI. All of the statistical tests were performed with a 95% confidence interval. We assessed the correlations of the BMI with the type of foot arch, gender, type of footwear worn, and prolonged position (more than two hours continuous per day) maintained using Pearson’s chi-square test at a 5% level of significance. We calculated the odds ratio to determine the prevalence of the various foot arches across the BMI categories.

The 360 participants recruited for the study had a mean age of 25.42 years. Out of these participants, 156 had low arches and 141 had normal arches; 12 had high arches bilaterally; and 51 had different arches in the left and right foot (Table 1). The descriptive characteristics of the participants listed in Tables 2, 3 present the association of BMI, gender, type of footwear, and prolonged position with the right and left foot arch type. Table 4 presents the distribution of BMI categories by gender. Table 5 presents the odds ratios suggesting the prevalence of high and low right foot arch in underweight, overweight, and obese subjects. Table 6 presents the odds ratios suggesting the prevalence of high and low left foot arch in underweight, overweight, and obese subjects.

**Table 1:** Arch type with number of participants

Type of Arch	Number of Participants
Bilaterally Low	156
Bilaterally Normal	141
Bilaterally High	12
Variation bilaterally	51

**Table 2:** BMI categories with mean and SD NTNH: Normalized truncated navicular height

NTNH of the BMI Categories	Mean ± SD	
	Left	Right
Normal	0.21 ± 0.045	0.21 ± 0.044
Underweight	0.23 ± 0.044	0.23 ± 0.048
Overweight	0.21 ± 0.046	0.21 ± 0.042
Obese	0.21 ± 0.05	0.21 ± 0.05

**Table 3:** Association of the test variables, chi-square test values, and p-values

Test Variable	Chi-Square Value	p-Value
BMI – Left Foot Arch Type	10.745	0.097
BMI – Right Foot Arch Type	9.746	0.136
Gender – Left Foot Arch Type	20.004	< 0.001
Gender – Right Foot Arch Type	15.836	< 0.001
Footwear – Left Foot Arch Type	13.808	0.008
Footwear – Right Foot Arch Type	9.657	0.047
Prolonged Position – Left Foot Arch	3.106	0.212
Prolonged Position – Right Foot Arch	1.838	0.399

**Table 4:** Distribution of BMI categories across gender

BMI Categories	Total	Male (%)	Female (%)
Obese	80	21 (6%)	59 (16%)
Overweight	80	20 (6%)	60 (17%)
Normal	120	29 (8%)	91 (25%)
Underweight	80	33 (9%)	47 (13%)

**Table 5:** Odds ratio suggesting the prevalence of high and low right-foot arch in underweight, overweight, and obese subjects

Characteristic Unit (Right Foot)	Odds Ratio (95% Confidence Interval)	p-Value
High Arch	Underweight: 2.54 (0.58–11.23)	0.21
	Obese: 0.46 (0.05–4.59)	0.59
	Overweight: 2.1 (0.4–11.07)	0.71
Low Arch	Underweight: 0.95 (0.53–1.7)	0.07
	Obese: 0.87 (0.49–1.54)	0.49
	Overweight: 1.8 (1–3.24)	0.24

**Table 6:** Odds ratio suggesting the prevalence of high and low left-foot arch in underweight, overweight, and obese subjects

Characteristic Unit (Left Foot)	Odds Ratio (95% Confidence Interval)	p-Value
High Arch	Underweight: 2.02 (0.46–8.97)	0.22
	Obese: 0.44 (0.04–4.35)	0.09
	Overweight: 1.89 (0.36–10)	0.67
Low Arch	Underweight: 0.61 (0.34–1.09)	0.09
	Obese: 0.79 (0.45–1.41)	0.54
	Overweight: 1.43 (0.79–2.59)	0.81

## Discussion

The aim of this study was to observe the height of the MLA in healthy adults across the BMI categories of the 360 participants in the study. In this study, 309 had equal arches bilaterally, of which 45.63% were normal, 3.88% were high, and 50.48% were low. 51 participants had different arches in their feet, of which 18 (35.29%) had a combination of a normal left side arch and a low right side arch, and 33 (64.7%) had a low left side arch and a normal right side arch. The p-value for the association of the BMI categories and right foot arch type was 0.097, and that for the BMI categories and left foot arch type was 0.136. These results suggest that there was no statistically significant association between the BMI category and the arch type.

Zhao et al. found an association between BMI and the arch height index, specifically individuals with relatively high BMIs had relatively high arch height index values [14]. These researchers concluded that individuals with higher body weights have comparatively larger and flatter feet and, hence, larger foot parameter values than individuals with lower body weights. Other studies have found that the values of the arch index increased incrementally from normal weight to obese individuals and attributed the greater arch index values in the obese group to a trend of comparatively flat feet in obese individuals [19-21]. In contrast, we found no association between BMI and the type of foot arch. Wearing et al. explained that the adiposity associated with relatively large BMI values may cause distortion of the footprint by increasing the mid-foot contact area without altering the bony configuration of the medial arch. A possible reason for the differences between the results of this study and those of other studies is the variation in measurement methods. The present study used NTNH method, whereas previous studies have used radiographic techniques, 2D footprint measurements, or scanner-based methods. In addition, this study included a large sample size (n = 360), while other studies reported small samples (ranging from 80 to 100) and more ethnically diverse populations [22].

According to Sahdegi et al., mobility and stability are the essential components when performing any lower limb activity [23]. The mobilizing or preferred foot performs most of the fine motor activities for mobility, whereas the stabilizing or nonpreferred foot provides stability during ongoing activities. Zifchock et al. explained that because most activities require the dominant foot, the nondominant foot functions to balance the body [24]. As a result, the nondominant foot experiences more loading, which leads over time to ligament laxity and a lower arch index than in the dominant foot. A further explanation for this difference between the feet may be that, because most of the population is right dominant, the left side is considered the nondominant side, so the values of right arch height may be greater than that of the left, resulting in higher NTNH values in the dominant foot (i.e., the right foot) compared with the nondominant foot (the left foot) [25].

This reasoning is consistent with our finding that 31 subjects out of 51 subjects with different arches in the left and right feet had a lower left arch. It is necessary to consider lower limb dominance, which we did not, a limitation of this study. The issue of foot dominance could be further explored by calculating the differences in the arch heights in a sample with an even distribution of left-dominant and right-dominant participants.

We also found a statistically significant association between gender and left and right foot arch type (p < 0.001), with female participants having lower arches than the male participants. Previous studies have found that women tend to have lower arches than men and attributed this finding to either physiological characteristics or sexual dimorphism. Physiological features of women such as greater ligamentous laxity, greater range of motion, lower muscular strength, increased Q angle, a tendency to develop genu valgum leading to foot pronation, a leaner body mass, and, again, sexual dimorphism (including a wider pelvis) contribute to their smaller and narrower feet and affect the morphology of the MLA [26-28]. Wunderlich and Cavanagh reported that the male subjects in their study had longer and broader feet than the female subjects and that there were many morphological differences between the feet of women and men, particularly at the arch, the “lateral side of the foot, the first toe, and the ball of the foot”; thus, the women had a wider forefoot, shorter metatarsals, and lower arches than the men [29]. In contrast with the findings of this study, Aurichio et al. found that though women exhibited a higher prevalence of obesity and had flatter feet than men, obese men exhibited pronated feet because of the impact of increased body mass on foot morphology [30]. However, their results could be due to the unequal gender distribution in their study (71% female and 29% male participants). Hence, a study involving an equal number of men and women might clarify the relationship between gender and the type of foot arch.

The limitations of this study include the unequal gender distribution and not taking into account lower limb dominance and ethnic background. Accordingly, we recommend that a similar study be conducted with an equal distribution of subjects who have similar types of arches and an equal distribution of the various BMI categories that takes into account lower limb dominance.

## Conclusions

The present study found no statistically significant difference in MLA height across the BMI categories. We did observe a significant correlation between gender and foot arch type and between the type of footwear and foot arch type, but no significant correlation was found between maintaining a prolonged position and foot arch type. We also found that high arches were more prevalent in the underweight BMI category group, while low arches were more prevalent in the overweight group and the obese group.
